# Supplementary material for: Rhabdomyosarcoma Associated with Core Myopathy/Malignant Hyperthermia: Combined Effect of Germline Variants in RYR1 and ASPSCR1 May Play a Role
Source: Genes (Basel). 2023 Jun 27;14(7):1360. doi: 10.3390/genes14071360 (PMC10378851; doi:10.3390/genes14071360)
Supplement: Supplementary file 1 [file genes-14-01360-s001.zip › genes-2446078-supplementary.pdf]

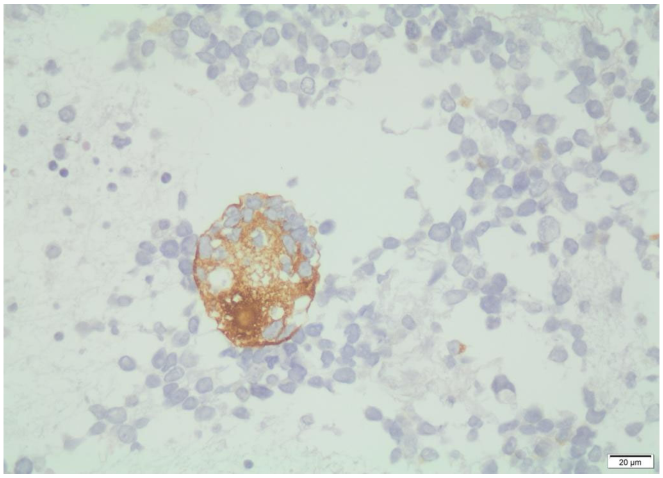

Supplementary Figure S1. Cytologic features of cervical nodule fine-needle aspiration cytology. Legend: Immunohistochemistry positive for desmin (focal labeling). Bar scale: 20 μm, 400×, Olympus BX50/Olympus SC100 microscope.
